# What did the dove sing to Pope Gregory? Ancestral melody reconstruction in Gregorian chant using Bayesian phylogenetics

**DOI:** 10.1371/journal.pone.0350014

**Published:** 2026-05-29

**Authors:** Gustavo A. Ballen, Klára Hedvika Mühlová, Jan Hajič

**Affiliations:** 1 Institute of Biosciences, São Paulo State University, Botucatu, São Paulo, Brazil; 2 Institute of Musicology, Masaryk University, Brno, Czechia; 3 Masaryk Institute and Archive, Czech Academy of Sciences, Prague, Czechia; 4 Institute of Formal and Applied Linguistics, Charles University, Prague, Czechia; Max Planck Institute for Empirical Aesthetics: Max Planck Institut fur empirische Asthetik, GERMANY

## Abstract

An attractive goal in the study of Gregorian chant melodies is reconstructing unobserved melodies as they may have been transmitted along their history, especially as early chant notation does not capture pitch exactly. We propose doing this computationally using Ancestral State Reconstruction over phylogenetic trees. Bayesian phylogenetic trees have shown promise as a tool to study the evolution of chant melodies, by inferring a plausible topology of chant transmission. However, the inferred trees cannot be used as Ancestral State Reconstruction inputs directly, because they are undirected, and their branch lengths conflate time and evolutionary rate. We therefore first apply Divergence Time Estimation to separate them and represent the tree in a directed form on the time dimension. Using Ancestral State Reconstruction, we then obtain reconstructions of melodies for each of the ancestral nodes, in addition to their distribution in time obtained from Divergence Time Estimation, and thus recover a phylogeny of chant melody with a music-historical interpretation. We applied this method to the Christmas Vespers dataset, and compare the results against musicological knowledge and melodies reconstructed at Solesmes using methods of contemporary philology, which shows potential for reconstructing cultural transmission through time.

## 1 Introduction

Gregorian chant is the universal liturgical monody of the Roman Catholic church, a major part of Western music history, and a dominant musical tradition up until at least the early 17th century. Its moniker comes from the legend of pope Gregory I., who supposedly dictated Gregorian repertoire as sung to him by a dove of the Holy Spirit. After the 2nd Vatican Council in the 1960s, the role of chant diminished, but it remains highly standardized, with a global authoritative edition: today, apps exist that contain the texts and melodies to be sung at each point of the day’s liturgy (e.g., ChantTools, SquareNote, and Neumz)

However, in the manuscript tradition between approximately 800 and 1600, one rarely finds the melody of a chant written exactly the same in two different sources. Exact pitch notation spread only after 1050 AD, which is also the case for melodies encoded in the adiastematic neume system. The Gregorian melodies spanning all of Latin Europe for the better part of a millennium, were (despite the institutional conservation pressure of their sacred nature) diverse. There are long-open questions in chant scholarship about the nature of this diversity [1–4], supported by the Cantus database, Cantus Index [5], and its digital ecosystem (i.e., https://dact-chant.ca/, SIMSSA Cantus Ultimus https://cantus.simssa.ca/).

We consider the evolutionary perspective on chant, with a tree where nodes depict “common ancestors” and terminals, – despite the role of lateral transmission in cultural evolution [6] – justifiable. This is because the Gregorian tradition does follow from a significant transmission event when a Roman tradition was brought and disseminated across the Frankish empire after 754 [7, p.514–518], and the subsequent Gregorian chant manuscript culture strongly relied on memory [8,9] and copying [10,11] predominantly from a single authority. Methods for explicit phylogenetic networks, that is, structures that incorporate horizontal transmission in the form of hybrid edges in addition to the tree-like part of the phylogeny [12,13], are available mostly extending from the coalescent multispecies model [14] into the multispecies coalescent model with introgression [15]. Regardless of the implementation, whether using composite likelihood or a full Bayesian approach, these models require many loci to carry out inference of reticulations, often on the order of hundreds to thousands of independent loci. Therefore, the still modest amounts of partitions available in chant datasets preclude the application of such methods to this system in cultural evolution. Despite this current methodological limitation, our approach using phylogenetic tree inference under Bayesian inference has shown promise by inferring a plausible Bayesian phylogeny of chant melody [16], but the method stops short of providing a music-historical interpretation of the resulting phylogeny as it is unrooted, limiting its value to chant scholarship.

We propose an extended procedure for inferring from chant melodies a phylogeny with a more defined music-historical interpretation, and thus the value of its predictions can be evaluated more accurately. In contrast to the unrooted topology of [16], here we infer a rooted tree ordered along the temporal dimension, so that every inferred internal node is associated with a distribution in time, which in turn enables sampling ancestral melodies from the posterior. We build this enriched Bayesian phylogeny of the Christmas Eve vespers dataset. With a lack of known ground truth to compare the resulting phylogeny to, we must instead evaluate the result against musicological knowledge, including the 20th-century Solesmes editions of chant, which (partially) aimed to reconstruct “original” chant melodies that, according to legend, the dove whispered to St. Gregory.

In 2023, Bayesian phylogeny inference using the sequence of pitches in a set of aligned melodies as data has been proposed [16], which sampled the posterior distributions of the unrooted topology as well as branch lengths, which were unfortunately not easy to interpret in a music-historical context. This limitation has directly inspired our present work. There are works from the history of chant scholarship that show evidence for the existence of certain melodic dialects [1,3], which should in fact be expected due to the oral nature of chant transmission [11,17,18], even though the extent of this orality is unclear [19].

The evolutionary perspective on cultural evolution has been applied in music [20] as well as in other areas, for instance in linguistics [21], and human tool development such as knots [22] and palaeolithic artifacts [23]. A phylogeny of Gabon folk music patrimonies has been proposed [24], which highlighted the role of vertical transmission, compared to the focus on lateral transmission in evolutionary linguistics [6]. Bioinformatics tools have been used to characterize diversity in Japanese vs. English and US folk melodies directly [25], or electronic music [26]; most recently, Billboard songs have been studied using methods from evolutionary cancer genomics reformulated as a variational autoencoder [27]. We also must mention the Cantometrics project [28], which proposed a high-level evolutionary tree of folk music worldwide. This ethnomusicological perspective can justifiedly be applied to chant [29].

With the exception of [25], these works extract features from their data that are known from other literature to be salient for the research questions posed. However, for chant melody (as opposed to features related to tonality for later European music), such features are not known yet. Computational work on chant melody has so far tried segmentation [30–34], but despite indications of the formulaic nature of parts of chant repertoire [35,36], these experiments have not yet led to a satisfactory theory of chant melody. Thus, we must use the chant melodies directly, with the attendant requirement of directly comparable melodies from different sources.

From the overview of related work, it is clear that computational chant scholarship is gaining momentum. However, the gap between computational results and their music-historical interpretation remains wide [16,34]. This work aims to provide a method that significantly reduces this gap with predictions directly testable against concrete musicological knowledge.

## 2 Materials and methods

### 2.1 Data: The Christmas Eve vespers and the solesmes edition

The dataset of Christmas Eve vespers, used in [16], is so far the best that we have available with a set of fully transcribed directly comparable melodies from a diverse selection of 14 sources: two secular French, three Cistercian, three Benedictine, one Augustinian, a secular source from the Low Countries, and a set of later secular sources from Bohemia. The dataset was originally collected in order to study relationships between late medieval Bohemian sources with the data including transcribed melodies available in the Hymnologica database (http://hymnologica.cz/jistebnice), and we combined this data with all further melodies available for Vig. Nat. Domini vespers from the Cantus Index interface, in order to cover a broader European context. Because the repertoire in office sources is not entirely consistent across sources and our system aligns melodies directly, we had to select a subset of chants contained in as many sources as possible, and then reduce the set of sources to those that contained as many of these chants as possible. The resulting dataset contained 14 sources, with fully transcribed melodies for the following Cantus IDs: 001737 (*Orietur sicut sol*, antiphon), 002000 (*Cum esset desponsata*, antiphon for the Magnificat), 003511 (*Judaea et Jerusalem*, antiphon), 004195 (*Bethleem non es minima*, antiphon), 007040a (*Constantes estote videbitis*, responsory verse), and 007040 (*Judaea et Jerusalem*, responsory). The responsory Judaea et Jerusalem has previously been misreported as Cantus ID 605019, which refers to a shorter version of the reponsory which uses the text “*Cras egrediemini*...” as a verse instead of respond, while 007040 uses “*Constantes estote*...” as the verse (Cantus ID 007040a). A full overview is given in Supplementary Table S1 in S1 File.

We originally intended to use the CantusCorpus dataset [30] of Office melodies. However, the authors of the Cantus Database preferred transcribing entire sources, so while there are more than 13000 fully transcribed antiphons in CantusCorpus v0.2 [31], the vast majority comes from less than 20 sources. This is further compounded by the surprising diversity of office repertoire. Thus, in the entirety of CantusCorpus, it is only possible to find 10 different sources that have transcribed melodies for the 5 antiphons. Hence, we decided to use the Christmas dataset, with its advantage of having been collected specifically in order to make the comparison between different sources possible.

For each of the 14 sources, we briefly report its century of origin, its provenance, and which ecclesiastical institution it belonged to (Supplementary Section S2 in S1 File).

The three major dimensions of external similarity between chant sources, in terms of how similar the segments of culture represented in these sources are expected to be, are geography, chronology, and *cursus*, i.e., space, time, and the liturgical context within which the books were used. It is not entirely clear in chant scholarship how strongly each of these factors should influence chant melodies (the exception where *cursus* is clearly expected to dominate other factors is that of the Cistercian order, which mandated that all monasteries must have identical liturgical books [37, p. 99]), but these organizing principles should be observed in the resulting tree.

Solesmes editions are found in the Gregobase corpus (https://gregobase.selapa.net), from where we collected the melodies corresponding to our inference dataset. However, only the responsory *Judaea et Jerusalem*, its verse *Constantes estote videbitis*, and the antiphons *Judaea et Jerusalem*, *Orietur sicut sol*, and *Cum esset desponsata* are available.

#### 2.1.1 Preprocessing.

Some sources contained multiple instances of chants of one Cantus ID: In that case, we retained the version with the most complete version of the melody (as repeated instances of the same chant are sometimes only written as incipits in the sources), and if multiple full melodies were available, we selected the melody that was directly in the Vig. Nat. Dom. section (Supplementary Table S1 in S1 File).

Furthermore, as noted by [34], differentiae (see https://differentiaedatabase.ca/about for details) tend to be encoded together with antiphon melodies and must be removed. Furthermore, in the Cistercian sources, some melodies had been recorded transposed a fifth up. This was a scribal practice based in the Cistercian reform of chant theory which has no implication on performance [38], so we transpose these melodies back by a fifth down, in order to not introduce spurious differences.

### 2.2 Methods

We extended an earlier procedure [16] that aimed to reconstruct the unrooted phylogenetic tree of sources by adding three additional steps: Model selection for the root using Bayes factors, divergence time estimation on the fixed rooted tree selected in the previous step, ancestral melody reconstruction using the resulting timetree, and finally comparison of the maximum a posteriori melodies with those reconstructed in the Solesmes edition of chant (Supplementary Figure S3 in S1 File).

A Bayesian phylogeny [39] of chant melodies was carried out following the approach by [16] using mrbayes_volpiano [16,40] (available at https://github.com/gaballench/mrbayes_volpiano). In short, the method uses the Mk evolutionary model [41] for an arbitrary number of discrete states to calculate the branch lengths in units of expected numbers of substitutions per site, which is in essence an evolutionary distance, and co-estimates the tree topology using the phylogenetic likelihood function [42]. The resulting tree is unrooted and has limited interpretation in terms of cultural transmission, because it is not clear how time flows along the tree (as opposed to rooted phylogenetic trees). Also, branch lengths in unrooted trees are measured in units that conflate evolutionary rate and time [43]. We identify rate and time using divergence time estimation methods [44] (see below) to measure evolutionary rate and its heterogeneity along the tree, and also to use time as the unit of branch lengths [14]. At that point we chose a single tree on which to carry out further analysis, and then calculated a maximum clade credibility tree as a summary tree from the posterior distribution of topologies, for which we used treeannotator [45]. Tree formatting was carried out with phyx [46].

#### 2.2.1 Model selection for the position of the root.

There is no obvious way to root a Gregorian chant phylogeny as it appeared as a unique event in time with no outgroups that we can explicitly use in phylogenetic analysis, at least not when one considers notated sources with comparable repertoire. A good choice would be some peripheral liturgy, but unfortunately the closest such candidate – Ambrosian/Old Roman chant – does not have comparable repertoire for the liturgical positions available in our dataset (and in any case its evolutionary relationship to Gregorian repertoire is unclear) [7, pp. 530–539].

Because we lack any indication of an outgroup with sequence data for analysis we can consider all possible root positions as fixed models and then calculate the marginal loglikelihood (*lnL*) using stepping stones under a Bayesian framework [47]. This approach allows model selection using Bayes factors ($BF_{i}=e^{lnL_{i}-max(lnL_{M})}$ where *i* is a given model out of *M* alternatives) while keeping the age information, the data, and other model parameters identical across analyses. An unrooted binary tree with *N* terminals has 2*N* − 3 branches, and therefore we potentially can root on each of them as the varying part of the model on these M=2N−3 model settings. Once picking the best root position via BF and model posterior probability ($PP_{model}=\frac{BF_{i}}{\sum_{i}^{M}BF_{i}}$), we can use the best rooted topology to carry out divergence time estimation (DTE).

Generation of all possible rooted trees on the MCC tree was carried out using the packages ape [48] and phytools [49] in R v.4.3 [50]. We applied stepping stones in mrbayes_volpiano with 50 stones and 1.000.000 generations sampling every 100th generation. The marginal likelihood was then used for calculating BF and model PP in R.

#### 2.2.2 Divergence time estimation (DTE).

Once we have selected a rooted tree Ψ we can use Bayesian DTE on the fixed topology in order to separate evolutionary rate *r* from time τ in branch lengths [51]. The Bayesian model in general form (Equation 1) uses time calibration densities f(τ) on either the nodes or the tips in order to generate the joint time prior f(τ,Ψ) using the calibration densities (Supplementary Table S4 in S1 File) and the tree process. Separation of rate and time requires also a clock model f(r|τ,θ) which describes the branch-specific evolutionary rates. The model parameters θ include parameters of the tree process, the clock model, and the evolutionary model. Then, the model estimates the PP densities for all of the model parameters conditional to the melody alignment data *D*.


$$\begin{array}{c} f(\tau ,r,\theta |D)=\frac{f(\theta )f(\tau )f(r|\tau ,\theta )f(D|\tau ,\theta ,r)}{f(D)} \end{array}$$ (1)

It is possible to extend the Bayesian model by specifying the tree prior using a fossilised birth-death model parametrised using speciation, extinction, and sampling proportion (equation 1 in [52]). These parameters are often difficult to model from previous knowledge, but it is possible to use “uninformative” or flat priors for them [53].

**Fig 1:**
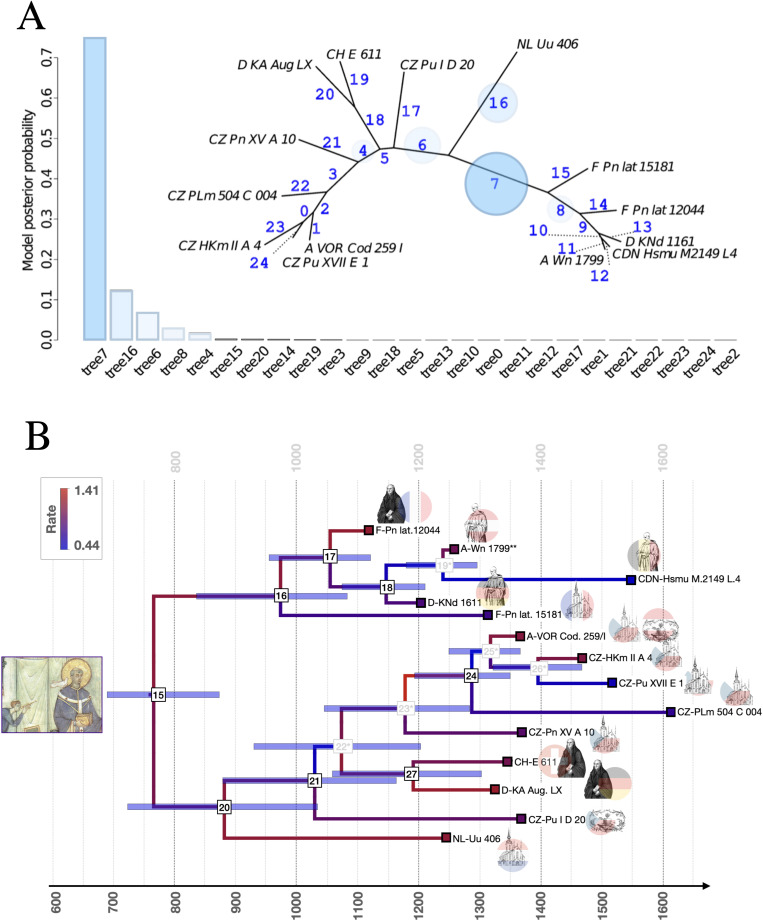
A) Model selection for the position of the root. Bar height represents posterior model probability, each bar is a topology resulting from rooting at a given branch in the maximum credibility tree. Rooted trees labeled tree0 through tree24. **B)** Divergence Time Estimation. The horizontal axis is time in years (A.D.). The blue horizontal bars at internal nodes represent the 95% highest posterior density intervals for divergence times. The color of the branches corresponds to evolutionary rate. Tips (colored squares) correspond to sources from the Christmas dataset, identified by their sigla. Their region of origin is encoded by a rondel corresponding to the current country governing said location, their cursus by the image (secular – church building, Benedictine – black monk, Cistercian – white monk, Augustinian – heart). The root separates a distinctly French and Cistercian clade (the order was founded in France) from sources east of the Rhine, following [3]. The grey internal nodes with asterisks are not in the majority consensus tree, highlighting topological uncertainty. Finally, a depiction of the Gregorian legend of the dove dictating melodies to Gregory I. from the Registrum Gregorii (Trier, Stadtbibliothek, Hs. 171/1626) is centered at the time of his papacy (590-604 AD).

DTE was carried out in mrbayes_volpiano using the Mk model with inverse-gamma rates [41]. The rooted tree topology was fixed. The clock model was set to independent gamma rates [54] and an Exponential(2) prior used for the variance, whereas an Exponential(5) prior was used for the clock rate. The FBD parameter priors were set to uniform(0,10) for the speciation rate, and Beta(1,1) for both the extinction and fossilisation rates. Please note that these rates are not necessarily normalized proportions and need not to be constrained to the interval [0,1], and are instead in units of number of speciation or extinction events per unit of time. Calibration densities were specified as in Supplementary Table S4 in S1 File. Parallel-tempering MCMC [55] was used with eight chains, one cold and seven hot, with a temperature of 0.001. MCMC sampling was carried out with 10.000.000 generations, sampling every 1.000. Posterior summarisation used a burn-in of 10%. All parameters attained effective sample size (ESS) > 500, indicating an appropriate sampling of the parameter posterior distributions. Time tree plotting (Fig 1) was carried out in figtree [56].

#### 2.2.3 Ancestral state reconstruction (ASR).

These methods infer most likely state of the sequence (position-wise) on the resulting posterior tree. This needs a fully resolved tree, so instead of the summary tree we use the maximum credibility tree. It is possible to generalise the independent ASR for each character in the alignment and then to collect the reconstructed states to represent a whole sequence, or in our case study, a melody, therefore producing an Ancestral Melody Reconstruction (AMR). ASR requires an evolutionary model for discrete states which in this case is the same the one used for phylogenetic inference (i.e., the Mk model).

Now we can use the Mk model for calculating the probability of transition as a function of time [51,57]. Here time are the branches, and we start from the observed states at the tips and traverse the tree towards the root estimating the ancestral states. Simulation is used to sample form the PP density for each of the states using stochastic mapping [58]. Because we have PP densities for each melody position at each internal node, we can calculate posterior probabilities for arbitrary melodies. For instance, we can take an aligned melody generated by another reconstruction method and calculate the probability of observing it at any arbitrary node of the tree under the PP density of states.

AMR was carried out using the equal-rates model in phytools [49]. Stochastic character mapping was used for constructing the posterior distribution on each node using empirical Bayes using 1000 iterations. This process required parallelizing the ASR for each position in the melody using the packages doParallel [59] and foreach [60] in R.

The Solesmes melodies (see 0.5) were aligned to the original alignments used for tree inference using mafft [61] with the –keeplength argument so that the base alignments remain unchanged. We then collected the maximum *a posteriori* states in order to construct the AMR on each node. Finally used the posterior of states on each internal node in order to calculate the per-node PP for both the AMR and the Solesmes reconstruction. Note that when producing MAP estimates of melodies using ASR, sometimes there is a tie at a certain position. This can mostly be resolved by preferring a note over a gap. In the rare case when there is a tie between two notes, we arbitrarily prefer the lower note.

#### 2.2.4 The Solesmes edition.

The comparative approach of Solesmes means they should not be included in building the phylogeny as the assumption about an evolutionary process does not hold, because such melodies did not evolve naturally through history but represent musicological reconstructions instead. However, because we have for each internal node a posterior distribution over melodies, we can compute the probability of observing the given Solesmes melody on each inferred node. Finally we calculated the joint probability of observing all the Solesmes melodies at a given node.

## 3 Results

The topology of the phylogeny on the Christmas dataset follows the same contours of geography and c*ursus* as the phylogeny of [16]. This is expected, as the alignment is similar and the tree inference methods are same. However, as the dataset has been cleaned, there are a few changes in the topology. There is a distinct French and Cistercian branch (given that the Cistercians originated in France, this is not surprising), then a geographical gradient towards the group of secular Bohemain sources (CZ and A-VOR), with a monastic group of Benedictine and Augustinian sources (CZ-Pu I D 20, D-KA Aug. LX, and CH-E 611). The historical Augustinian link between CZ-Pu I D 20 and A-VOR 259 I is, however, not replicated.

### 3.1 Evolution of melodies in a temporal scale: DTE

#### 3.1.1 Root position.

A single rooted tree was found to be the best one (*lnBF* compared to the next best tree 1.8; model PP = 0.75) and used for subsequent analysis. The model selection recovered the root position so that the highest-level clades correspond to French and Cistercian sources and non-Cistercian sources coming from the eastern side of the river Rhine in the other clade (Fig 1). The Cistercian order was founded at Citeaux, an abbey in France, and the order was strict about maintaining this lineage also for chant books. This corresponds to existing hypotheses about the distinct “west Frankish” and “east Frankish” chant melodic dialects [3].

### 3.2 Estimating ancestral melodies: AMR

**Fig 2:**
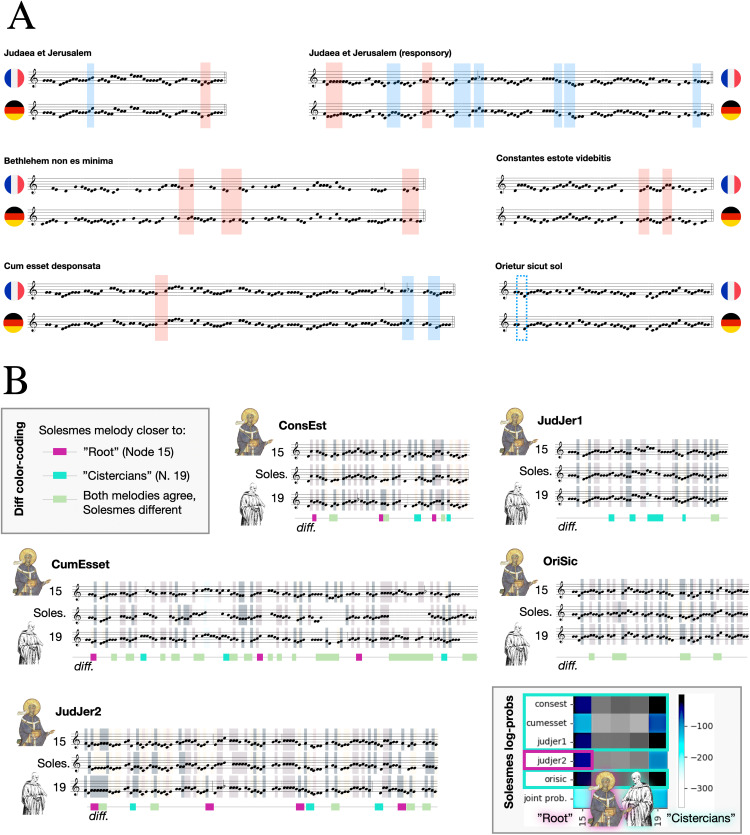
A) Comparing the results of Ancestral State Reconstruction for the nodes corresponding to the common ancestors of the East Frankish (“Germanic”, node 20) and West Frankish (“French”, node 16) melodic dialects. Each pitch is assigned a background color to make differences in melodies easier to spot. In previous literature on the topic (Peter Wagner’s book “Gregorianische Formenlehre”, 1911), the “Germanic” dialect was supposed to be characterized by its preference of *fa* over the “French” dialect’s preference for *mi*, which corresponds to the “Germanic” dialect preferring the tone *f* over *e* and *c* over *b* (natural, also termed “h” in German nomenclature, as well as flat), which given the prevalence of stepwise motion often translates to preferring thirds *d-f* and *a-c* over seconds *d-e*, *e-f*, and *a-b(h)*, *b(h)-c*. While the sources were “sorted” into these two main melodic dialects by the phylogeny, this preference seems to very weak in the inferred ancestral melodies for these two dialects: while in 7 positions the East Frankish dialect does exhibit this preference (**green** frames), in 3 positions this is the other way around (**red** frames), and most importantly, there are many places where the East vs. West Frankish preferences could have been expressed but is not (some examples in **blue**). **B)** Analysis of the reconstructed melodies for the inferred root (node 15 of the DTE tree), the common ancestor of the later Cistercian sources (node 19 of the DTE tree), and the Solesmes version of the melodies. The log probability of observing the Solesmes melody at the Cistercian node is higher for each of the five melodies (the antiphon *Bethleem non es minima* is not in the Solesmes editions) except for the responsory Judaea et Jerusalem. Under each of the melody comparisons, we color-code the positions where Solesmes agrees with the root and not with the Cistercians (**purple**), positions where Solesmes agrees with the Cistercians rather than the root (**teal**), and positions where both inferred melodies agree but the Solesmes edition differs (**light green**), against the MAP estimates of the melodies obtained from ASR. Most of the differences are between the Solesmes edition and both of the inferred medieval melodies: thus one can observe where the Solesmes editorial process prioritized other criteria than fidelity to the earliest possible sources.

Given that the root position found via model selection separates the tree into east Frankish “Germanic” (node 20) vs. west Frankish “Romanic” (node 16) melodies [3,4], we focus on comparing the melodies inferred for the roots of these two clades. The “Germanic” chant dialect has been characterized by its preference for minor thirds d-f, a-c instead of seconds e-f, a-b (incl. a-b flat) [4], with the insight that it is a general German preference for *re*-*fa* over **re**-*mi* or *mi*-*fa*. A melody in a “Germanic” source exhibiting none this preference merits scrutiny [4, p. 95] (Fig 2).

This is observed in the inferred melodies to a limited extent. For five out of six Cantus IDs in the dataset, at least one such position where node 20 preferred what is in [3,4] considered Germanic. At the same time, four melodies also have positions where the “Romanic” clade root MAP estimate has “Germanic” features. Out of a total of 77 manually identified “opportunities” for the melodies to differ in their treatment of *re*-*fa* thirds, the “Germanic” option is only chosen by node 20 melody 5 more times than by the node 16 melody. This may partly be an artifact of mode. Most of the Christmas melodies are in mode 8, while the most characteristic examples in [3] and [4] are in mode 1. However, the literature does also indicate that the melodic dialect doesn’t at all manifest at every opportunity, and that one might easily find the “Germanic” preference in sources west of the Rhine and vice versa [4, pp.95–98]. Therefore, while ASR results are not as clear a success as accurately predicting the timing of the Cistercian reformation, it does again conform to existing expectation.

### 3.3 Comparing AMR to the Solesmes editions

The posterior log-probabilities of observing the Solesmes melodies at individual nodes are shown in Supplementary Figure S5 in S1 File. At least for the Christmas Eve vespers, the model indicates the editors of Solesmes have preferred melodies of the west Frankish style (nodes 16–19), most prominently those of the Cistercian order (nodes 18 and 19). The melodic features of later secular Bohemian sources (nodes 24–26) are the least preferred models overall, which is mostly influenced by the longer responsory *Judaea et Jerusalem* (judjer2) and the Magnificat antiphon *Cum esset desponsata* (cumesset), not so much by the psalm antiphon *Orietur sicut sol* (orisic) or the responsory verse *Constantes estote* (consest).

This complements what we know about the process, as well as expectation of the time [1, pp.5–6]. The Solesmes editorial process did not, for unexplained reasons, consider German sources [4, p.113]. Furthermore, the second and third criteria – legitimate tradition and needs of contemporary liturgy, which included the skill sets and habits of early 20th century singers [62,63] – would gravitate towards a later aesthetic of chant that is closer to the world of tonality, and the Cistercian reform is a significant stepping stone in this respect [38].

## 4 Discussion

We introduce a method for building interpretable phylogenies of chant melody. Because there is no known “true” phylogeny of chant, we rely on comparing the resulting phylogeny to music-historical knowledge. The experiments on the cleaned Christmas dataset and with the Solesmes melodies have provided a number of predictions that could be directly verified against existing knowledge (musicological, codicological, paleographical, historical) and, in this confrontation, have held. The accuracy of the estimated median age of the common ancestor of Cistercian sources is notable. We believe that we have significantly narrowed the gap between computational methods and musicological insight, and that this method – especially when applied to larger datasets, or datasets designed explicitly around open problems – can meaningfully complement philological methods of chant research.

### 4.1. Cistercians sources

There are three sources in the Christmas dataset of Cistercian provenance: A-Wn 1799**, D-KNd 1611, and CDN-Hsmu M2149.L4. The Cistercian order is known to have maintained very strict discipline in copying liturgical books. This is already seen in the topology inferred in [16], where these sources are grouped closely together, and this is replicated in our topology as well. However, additionally, the DTE step of our pipeline provides an explicit time for the common ancestor of Cistercian sources: the median falls on the year 1145, which coincides with the reform of the antiphonary initiated by St. Bernard of Clairvaux in 1147 or prior [64]. Drafts of the Bernardine liturgy are found in the 12A-B Westmalle Antiphonary, dating from 1140–1143 [38]. The 95% credibility interval covers 1075–1210.

### 4.2 The position of A-VOR Cod. 259 I

The source A-VOR Cod. 259 I merits attention. It is a source from the late 14th century that was brought to Vienna and later the Augustinian monastery in Vorau when the Chapter house at Vyšehrad (Prague) was fleeing the Hussite wars of 1420–1434, as evidenced by the inscription on its f.1r. This inscription, which describes the history of the manuscript, is written over a rubbed-out earlier melody (Gaude et laetare, Cantus ID 002922, which is often the first antiphon in numerous sources of Bohemian provenance), which is how the new owners found space for the inscription on the first page. Why is this source not grouped closer with either the Augustinian CZ-Pn I D 20, or the similarly late 14th century CZ-Pu XV A 10? In 1496, the manuscript underwent significant revisions according to the Salzburg diocese rite [65]. A look at its digitized folios 69r-71v reveals the presence of possible palimpsests (instances of an older layer scratched out: see https://www.cantusplanus.at/common/rism.php?rism=A-VOR259_1). Some are clearly visible (the doxology at the top of f.71r), and some are just suspicion (traces of rhombic shapes that resemble bleedthrough in the last two staffs on f.69r but do not have corresponding rhombes on f.69v or the neighboring f.68v, which incidentally also has modifications in its bottom half). Furthermore, the antiphon “Gaude et laetare”, overwritten on f.1r by the inscription with the source’s history, is used again on f.70r for Christmas Eve vespers, but with a different melody than what was on f.1r – another reason to suspect that the melodies for Christmas Eve vespers may have been changed later in the 15th century. Of course, codicological expertise is needed to resolve the extent to which this was done; however, this shows how the inferred phylogeny can, aside from providing verifiable predictions such as the Cistercian common ancestor date, also point towards specific problems requiring further musicological study.

If we do accept the later origin of A-VOR Cod. 259 melodies, the branch leading down to node 24 that exhibits the highest evolutionary rate can be symptomatic of two phenomena: one geographic, the Bohemian Reformation, and one chronological – the transition into the late middle ages. The fact that the A-VOR melodies were modified outside of the lands controlled by the Hussites (or, later, Utraquists) lends, in our view, more credence to the chronological explanation (which then leads to unexplored questions about chant melody between the late middle ages and the Editio Medicaea of 1604), but weighing these two interpretations is not possible with the Christmas dataset.

The case study of A-VOR Cod. 259/I shows that this method also brings value in reexamining assumptions about the input data, and thus can also serve as a “quality control” tool. Its improved interpretability further makes it more accessible for musicologists without a significant technical background.

### 4.3 The Solesmes edition and AMR

We now use our estimated model of chant evolution to study the modern chant restoration movement through the properties of the melodies from the group of 20th- and 21st-century editions of chant produced primarily by the Benedictine monks of the Solesmes Abbey. The Solesmes editions are the central output of the chant restoration movement, led by the Benedictine monks of Solesmes since the latter half of the 19th century [66]. Their core output is the official new edition of Gregorian chant – originally under the auspices of the Editio Vaticana, led by Dom. Pothier and an editorial council, based on the document *Motu Proprio* of Nov 22nd, 1903, of pope Pius X; later editorial activity has been carried out by the monks of Solesmes themselves.

The main contribution of this edition was, in line with the philological thinking of the time, to recover as much as possible the “original” melodies of Gregorian chant, trying to recover – in principle – what the dove had whispered into Gregory I.’s ear. Thus, in the context of ASR, it is enticing to use the Solesmes editions as the “best effort” reconstruction of the melodies that should correspond to the root of our tree, and thus different ways of building the pipeline could theoretically be evaluated by how similar their predicted root melodies are to the Solesmes edition – thus matching this ongoing “best effort” reconstruction using philological methods. However, reconstructing the earliest melodies of chant was not the only priority of Editio Vaticana: another was to do this while retaining “legitimate tradition” from later times, and third, to serve the needs of the day. From the *Motu Proprio* of Pius X.: “The Gregorian melodies are to be restored in their completeness and true nature, according to the testimony of the more ancient manuscripts, taking into consideration not only the legitimate tradition of intervening centuries, but also the common practices of present-day liturgy.” Transl. from [66, p.xxi]. The clashes between these priorities, as manifested in the resultant melodies, were subject to heated exchanges at the time [62,63,67]. and the process involved diverse influences such as anticlericalism or business interests of print unions [68]. This can be observed also in the melodies directly when we compare them with the inferred medieval melodies in Fig 2.

The melodies thus cannot be used to directly evaluate how well the root melody is reconstructed by ASR. Instead, observations can be made about the results of the Solesmes efforts: what was the extent to which these were “archaeological”, and what influence did “legitimate tradition” and “common practices of present-day liturgy” have? Here we borrow the term that Peter Wagner applied in his polemic with the critics of Editio Vaticana [63], while we do not necessarily agree with its original disparaging nature, it is not chosen without merit to represent that particular methodological approach. How does this recent layer of chant tradition relate to the medieval sources? These results serve to show how the inferred phylogeny can be used to provide insights complementary to philological methods, and perhaps also how the editorial practices of Solesmes evolved throughout the 20th and 21st centuries.

## 5 Conclusion

Further evaluation should be done on other data, such as the Officium Defunctorum study by Ottosen [69].

The method could then be applied to existing open problems in chant transmission, such as the issue of transmission of the “Germanic” melodic dialect [4, pp.116–117]. The fact that DTE predict a slightly earlier common ancestor for the east Frankish sources may be a part of the discussion, although more sources in the dataset are needed to try and narrow the time ranges down. Similarly, given an encoding of melodies from Old Roman sources, this method could provide additional empirical evidence of its chronology in relation to Gregorian chant, which is still an open problem [17, p. 466].

Another suggestion would be examining vernacular chant traditions, such as the early 15th century Jistebnice Cantionale [70] and later utraquist liturgical books in Bohemia [71], or modern editions of Chinese vernacular chant [72,73] or Korean chant [74].

Another open problem is early chant transmission in adiastematic sources. Here, phylogenies could utilize sequences of neumes and/or visual features of the notation, rather than the melodies themselves. The Music Enoding Initiative (MEI) standard supports adiastematic neumes, but manual encoding is costly and few have the expertise. However, as opposed to finding statistical patterns through machine learning, the phylogenetic pipeline has shown usefulness already with less than 100 transcribed sequences, making it a cost-effective option. Furthermore, by placing an adiastematic source into the phylogenetic tree, one can perhaps use the inferred ancestral melody to help resolve ambiguities of pitch when they arise.

Further work on Solesmes melodies may also bring historical chant research in a closer relationship to current living practice, which is based on these editions. Specifically, the activities of the schola in Kiedrich (see https://www.kiedricher-chorbuben.de/index.php/musik/choral) in reviving the Germanic dialect (with a papal dispensation), in combination with the Neumz project recorded at a French Benedictine abbey (see https://neumz.com/) can provide a stepping stone towards an ability to process the audio modality and start including features of performance practice in analyses. Using melody alone, independent of genre-specific features such as liturgical positions, also enables tracing melodic evolution across repertoires. Can geographically defined melodic dialects show commonality with, e.g., regional folksong traditions? Or, can we perhaps even find traces of mostly lost pre-Gregorian repertoires such as Gallican chant or the chant of the British Isles?

Whatever the future findings, we are excited for the new options that this phylogenetic pipeline brings to the field of not only computational chant scholarship.

## Supporting information

S1 File Supplementary materials S1–S5 are available in the supplementary file. (PDF)
