# Supplementary material for: What did the dove sing to Pope Gregory? Ancestral melody reconstruction in Gregorian chant using Bayesian phylogenetics
Source: PLoS One. 2026 May 29;21(5):e0350014. doi: 10.1371/journal.pone.0350014 (PMC13221024; doi:10.1371/journal.pone.0350014)
Supplement: S1 File — (PDF) [file pone.0350014.s001.pdf]

What did the dove sing to Pope Gregory? Ancestral melody reconstruction in Gregorian chant using Bayesian phylogenetics Supplementary material

Gustavo A. Ballen[1,*], Klára Hedvika Mühlová[2], Jan Hajič jr.[3]

[1] *Instituto de Biociências de Botucatu, Universidade Estadual Paulista "Júlio de Mesquita Filho", Botucatu, SP, Brazil, gustavo.a.ballen@gmail.com*

[2] *Institute of Musicology, Masaryk University, Czechia, muhlova@mail.muni.cz*

[3] *Masaryk Institute and Archive, Czech Academy of Sciences, Czechia, hajic@mua.cas.cz*

∗Corresponding author

1 Supplementary Table S1

**Source**	**Provenance**	**Date**	**Cursus**	**605019**	**001737**	**002000**	**003511**	**004195**	**007040a**
A-Wn 1799**	Rein	1200s	Cistercian	1	NA	1	1	1	1
A-VOR Cod. 259/I	Prague	1360	Secular	1	2	1	1	1	1
CDN-Hsmu M2149.L4	Salzinnes	1554	Cistercian	1	NA	1	1	1	1
CH-E 611	Einsiedeln	1300s	Benedictine	1	3	1	1	1	1
CZ-HKm II A 4	Hr. Král.	1400s	Secular	1	1	1	1	1	1
CZ-PLm 504 C 004	Plsen	1616	Secular	1	1	1	1	1	1
CZ-Pu XVII E 1	Bohemia	1516	Unknown	1	NA	1	1	NA	1
CZ-Pn XV A 10	Prague	1300s	Secular	1	1	1	1	1	1
CZ-Pu I D 20	Passau	1300s	Augustinian	1	1	1	1	1	1
D-KA Aug. LX	Zwiefalten	1100s	Benedictine	1	1	1	1	1	1
D-KNd 1161	Köln	1200s	Cistercian	1	NA	1	1	1	1
F-Pn lat. 12044	Paris	1100s	Benedictine	1	1	1	1	2	1
F-Pn lat. 15181	Paris	1300s	Secular	1	NA	1	1	2	1
NL-Uu 406	Utrecht	1150	Secular	1	2	1	3	2	1

Sources of the Christmas Vespers dataset. The provenance, approximate date, cursus, and presence of the chant in each source (1 or more instances per source). NA represents chants not present in a given source.

2 Supplementary Section S2

Details of the sources bearing melodies. For each of the 14 sources, we briefly report its century of origin, its provenance, and which ecclesiastical institution it belonged to.

- **A-VOR Cod. 259/I.** A 14th century antiphoner of the collegiate chapter church of Vyšehrad, Prague. In the early 15th century, it was moved to Vorau because of Hussite wars. In 1490-1500, it was adapted for Salzburg liturgy. Available at https://manuscripta.at/hs_detail.php?ID=6267.
- **A-Wn 1799**.** A 13th century Cistercian antiphoner from the Rein monastery in Austria. Available at https://differentiaedatabase.ca/manuscripts/wn-1799.
- **CDN-Hsmu M2149.L4.** Cistercian antiphoner from the Abbey of Salzinnes, Namur, in the Diocese of Liège, central Belgium, completed in 1554-1555. Available at https://cantus.uwaterloo.ca/source/123723.
- **CH-E 611.** A 14th-century antiphoner from the Benedictine monastery of Einsiedeln, central Switzerland. Available at https://cantus.simssa.ca/manuscript/123606/.
- **CZ-HKm II A 4.** An antiphoner from the 1470s, belonging to the municipal Church of the Holy Spirit in Hradec Králové, eastern Czechia. Available at http://hun-chant.eu/source/1481?page=1.
- **CZ-PLm 504 C 004.** A late antiphonary from the St. Bartholomew municipal church in Pilsen, western Czechia, from 1616. Available at https://rukopisy.zcm.cz/view.php?ID=504C004.
- **CZ-Pn XV A 10.** Late 15th century notated breviary from the cathedral cursus in Prague, Czechia. Available at http://hymnologica.cz/source/47.
- **CZ-Pu I D 20.** An antiphonary from the Augustinian monastery in Třeboň, southern Czechia, created in the 2nd half of the 14th century. Available at http://hymnologica.cz/source/10721.
- **CZ-Pu XVII E 1.** A mixed Latin and Czech antiphonary from the early 16th century, of Czech (but further unspecified) provenance. Available at http://hymnologica.cz/source/10664.
- **D-KA Aug. LX.** A complex 12th-century antiphoner, of which the musical notation was almost completely rewritten in the 13th or 14th centuries. From the Zwiefalten Benedictine monastery in southwestern Germany, moved to the abbey of Reichenau in the 15th century. Available at https://cantus.uwaterloo.ca/source/123612.
- **D-KNd 1161.** A late 12th- and early 13th-century Cistercian antiphoner, possibly written for use by the female abbey of Saint Mechtern in Cologne, western Germany, renamed Saint Apern in 1477. Available at https://cantus.uwaterloo.ca/source/601861.
- **F-Pn lat. 12044.** An early 12th-century antiphoner from the Benedictine abbey of St.-Maur-de-Fossés, close to Paris, France. Available at https://cantus.uwaterloo.ca/source/123628.
- **F-Pn lat. 15181.** An early 14th-century notated breviary belonging to the Notre Dame cathedral in Paris, France. Available at https://cantus.uwaterloo.ca/source/123631.
- **NL-Uu 406.** A 12th-century antiphonary from St. Mary's church in Utrecht, Netherlands. Later 13th-15th-century changes. Complex source that has multiple versions of some melodies. Available at https://cantus.uwaterloo.ca/source/123641.

3 Supplementary Figure S3

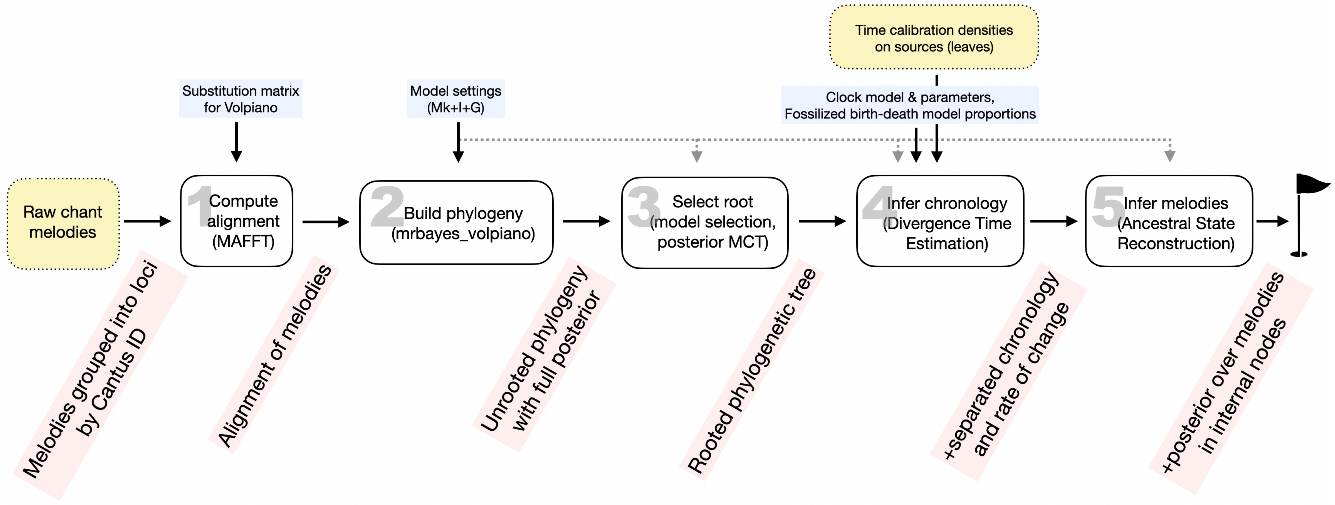

Overview of the whole pipeline. Externally provided data is highlighted in yellow, parameters in blue, artefacts at intermediate steps in red. Steps 1 and 2 have been described in [1], in this paper we focus on steps 3-5. These steps start from an unrooted phylogeny, which – while its topology already provides some insight into the development of chant melody – has no chronology and therefore no music-historical interpretation; and they lead to a phylogeny that assigns to all its inferred internal nodes a distribution in time and a posterior distribution over the melodies corresponding to that node, therefore making predictions that can then be verified against music-historical knowledge, and provide new insights as well.

4 Supplementary Table S4

Node	CD (YBP)	CD (AD)	Ref
A VOR Cod 259 I	654	1370	https://manuscripta.at/hs_detail.php?ID=6267
A Wn 1799	724–824	1200–1300	https://differentiaedatabase.ca/manuscripts/wn-1799
CDN Hsmu M2149 L4	474	1550	https://cantus.uwaterloo.ca/source/123723
CH E 611	624–724	1300–1400	https://cantus.simssa.ca/manuscript/123606/
CZ HKm II A 4	554	1470	http://hun-chant.eu/source/1481?page=1
CZ PLm 504 C 004	408	1616	https://rukopisy.zcm.cz/view.php?ID=504C004
CZ Pn XV A 10	624–674	1350–1400	http://hymnologica.cz/source/47
CZ Pu I D 20	624–674	1350–1400	http://hymnologica.cz/source/10721
CZ Pu XVII E 1	474–524	1500–1550	http://hymnologica.cz/source/10664
D KA Aug LX	624–924	1100–1400	https://cantus.uwaterloo.ca/source/123612
D KNd 1161	799–849	1175–1225	https://cantus.uwaterloo.ca/source/601861
F Pn lat 12044	874–924	1100–1150	https://cantus.uwaterloo.ca/source/123628
F Pn lat 15181	674–724	1300–1350	https://cantus.uwaterloo.ca/source/123631
NL Uu 406	624–924	1100–1400	https://cantus.uwaterloo.ca/source/123641
Root	1124–1324	700–900	[2]

Calibration densities (CD) used in DTE. Time scale is in both years before the present (YBP, as used by `mrbayes_volpiano`) as well as in anno Domini (AD). Single time values represent fixed values whereas intervals represent Uniform(min,max) calibration densities.

5 Supplementary Figure S5

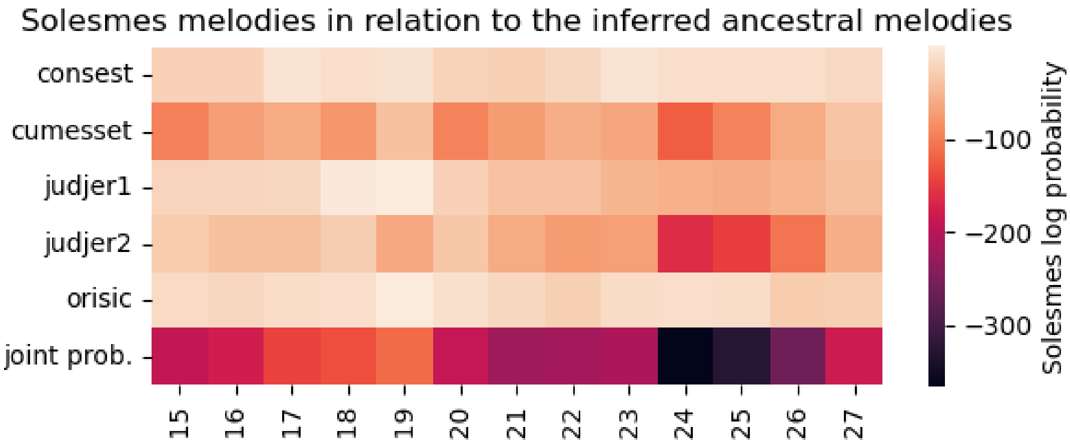

AMR of specific melodies compared to the Solesmes edition melodies. The log-probability of observing individual Solesmes melodies according to the posteriors inferred for internal nodes of the inferred phylogeny, and their joint probability. The node labels are taken from Fig. 1 in the main text: nodes 17, 18 and 19 correspond to the French and Cistercian monastic melodies, and the nodes with lowest log-probabilities are the Bohemian nodes. Melody names are *Constantes estote videbitis* (consest), *Cum esset desponsata* (cumesset), the antiphon *Judaea et Jerusalem* (judjer1), the responsory *Judaea et Jerusalem* (judjer2), and *Orietur sicut sol* (orisic).

References

[1] Hajič jr., J., Ballen, G. A., Mühlová, K. H. & Vlhová-Wörner, H. Towards Building a Phylogeny of Gregorian Chant Melodies. In *Proceedings of the 24th International Society for Music Information Retrieval Conference*, 571–578 (ISMIR, 2023). URL https://doi.org/10.5281/zenodo.10340442.

[2] Hiley, D. *Western plainchant: a handbook* (Clarendon Press, Oxford, United Kingdom, 1993).
